# Editorial: Xenobiotics and emerging contaminants in ecosystems: innovative geo-microbial strategies for prevention, efficient clean-up and biosafety

**DOI:** 10.3389/fbioe.2025.1619769

**Published:** 2025-05-20

**Authors:** Balaram Mohapatra, Avishek Dutta, Prashant S. Phale, Upendra Kumar

**Affiliations:** ^1^ Environmental Biotechnology Department, Gujarat Biotechnology University, Gandhinagar, Gujarat, India; ^2^ Department of Geology, University of Georgia, Athens, GA, United States; ^3^ Savannah River Ecology Laboratory, University of Georgia, Aiken, SC, United States; ^4^ Department of Biosciences and Bioengineering, Indian Institute of Technology Bombay, Mumbai, Maharashtra, India; ^5^ ICAR-Central Rice Research Institute, Cuttack, Odisha, India

**Keywords:** xenobiotics, emerging contaminants, microbial transformation, omics, targeted intervention, biosafety, biosecurity

**FIGURE 1:**
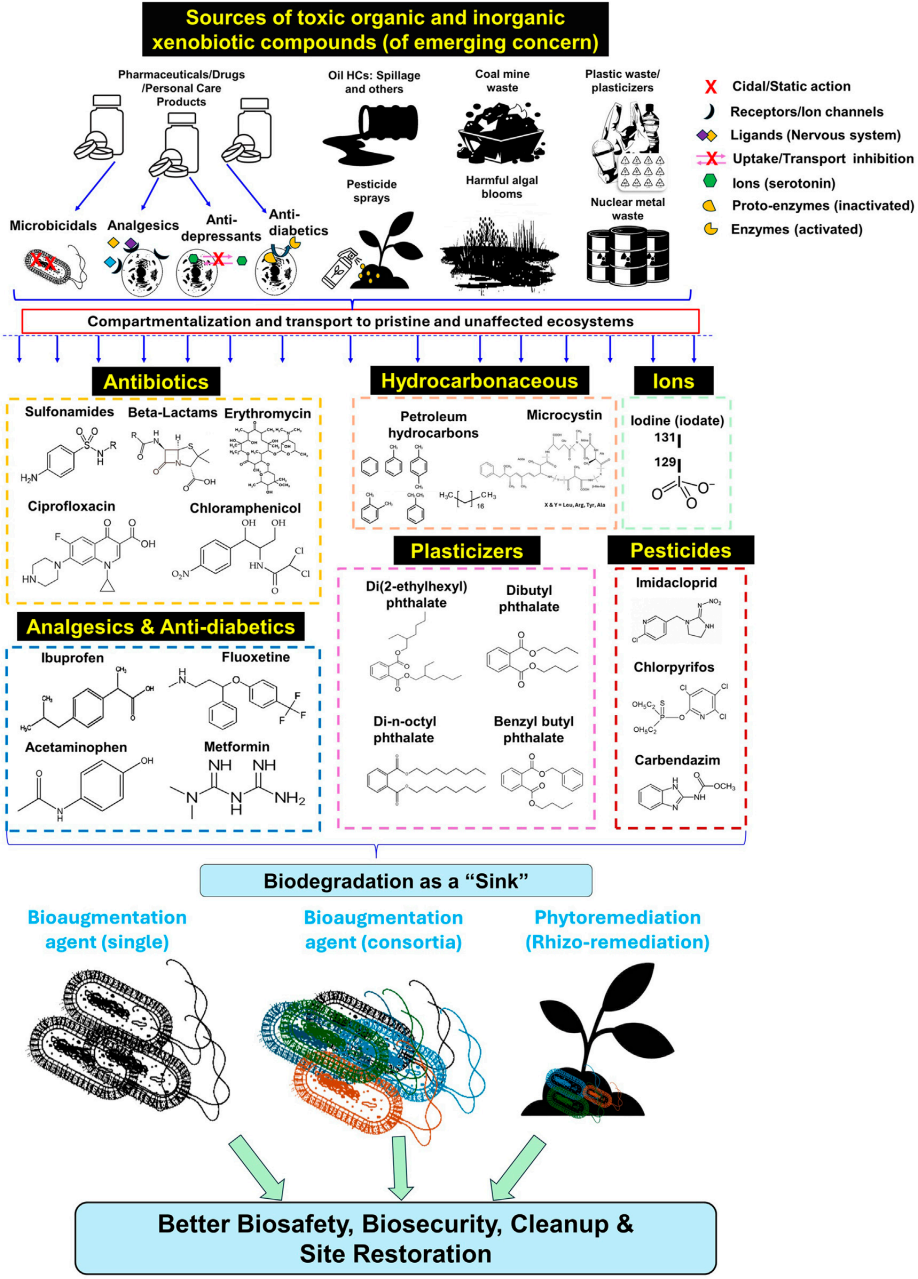
*Depiction* of various discharge routes/sources of toxic organic/inorganic xenobiotics (antibiotics, analgesics, anti-diabetics, cyanobacterial toxins, plastic derivatives/plasticizers, pesticides and metal/ions) into ecosystem strata and importance of biodegradation in the cleanup of these pollutants to achieve better biosafety/biosecurity.

Anthropogenic emissions and industrial discharge of toxic xenobiotic organic compounds, *i.e.*, (un)saturated hydrocarbons, aromatics (mono/poly-aromatics) and their substituted derivatives as historical contaminants, emerging- [e.g., pharmaceuticals and personal care products (PPCPs), micro/nano plastics, and nanomaterials], and forever -chemicals are creating dire ecological consequences (Summarized in Figure 1) (Mohapatra and Phale, 2022). The Environmental Protection Agency (EPA) has prioritized 129 of such compounds (acenaphthene being the first on the list) that are regulated under the Clean Water Act (https://www.epa.gov/sites/default/files/2015-09/documents/priority-pollutant-list-epa.pdf). The majority of these compounds are highly hydrophobic (with a high LogP_*ow*_), genotoxic, and mutagenic, and have endocrine disrupting- (cholinergic excess, hormone analogs, transport inhibitors, *etc*.) and cytotoxic-activities (form adducts, induce lipid peroxidation, impair CYP_450_ function, depurination, permeabilization of membranes, disruption in energy transduction, etc.) (Mohapatra and Phale, 2021). A few are classified as human carcinogens, *e.g.*, polychlorinated biphenyls (PCBs), and polychlorinated dibenzofurans (PCDFs), which have a higher bio-accumulative nature. These chemicals are found in various forms, such as aerosols, fumes, particulates, liquids, and deposits, and can transfer through percolation, runoff, advection, infiltration, and other secondary processes (coordination/co-complexation/co-precipitation, *etc.*). Thus, they severely impact the ecosystem’s biogeochemical functions (Gonzalez-Gaya et al., 2019). Alternatively, these xenobiotics exert selective pressure on the natural microbiota, prompting the evolution of adaptive and metabolic strategies to overcome their toxic effects, survive, and attenuate such effects at the impacted sites. However, various hurdles, such as environmental cues (lack of essential nutrients, inhibitory factors, and redox dynamics), microbial/cellular factors (availability of electron donors/acceptors, carbon repression, species competition, regulation of enzyme induction, eco-physiology traits, *etc.*), and physicochemical conditions (type, concentration, mass balance, and bioavailability) render the process ineffective on a large scale (Phale et al., 2020). Although the use of multi-OMICS technologies including meta-genomics (environmental DNA), transcriptomics (RNA), proteomics (cellular/sub-cellular proteins), metabolomics (metabolites), mobilomics (mobile genetic elements), and fluxomics (metabolic flux) combined with advanced bioinformatic tools has provided new insights into the underlying mechanisms of such field-scale bioremediation/containment, the extent of their efficacy (because of colonization resistance) and sustainability remains unanswered (Sharma et al., 2022). Traditional and new-age engineering bioremediation reactions based on synthetic biology/metabolic engineering have worked well under *in vitro*/discrete locations, but they have not yet been fully implemented at *in vivo*/large-scale/planet-wide settings, possibly due to rate-limiting biological/enzymological steps (de Lorenzo, 2022). Hence, a multi-sectoral effort to assess the eco-toxicology and biosafety of these compounds, preventing their entry into the ecosystem, and developing advanced-eco-friendly-*cum*-deployable abatement technologies and bioprocesses (*in-situ*/*ex-situ* and engineered) is necessary for effective cleanup (Kuppan et al., 2024; Visvanathan et al., 2024). Considering the urgent situation, this Research Topic focused on understanding toxic and hazardous xenobiotic emerging contaminants in ecosystems, along with innovative geo-microbial strategies for efficient cleanup and biosafety. A total of five manuscripts were submitted for this issue, one of which was rejected and four of which were published. This editorial article on this Research Topic emphasizes the effective strategies for cleaning up such xenobiotics, thus reducing the potential risk of exposure to biota.

According to Shah et al., microbe-mediated strategies (metabolic, genetic, and enzymatic) are of the utmost importance for the cleanup of contaminants of emerging concern (CECs: pharmaceuticals, personal care products, nanomaterials, pesticides, plasticizers, microplastics, cyano-/algal-toxins, and PFOS/PFAS). The detection of these compounds can be attributed either to their recent introduction into the environment or to advancements in detection technologies, but with poorly understood risk profiles (Podder et al., 2021). However, microbial candidates are able to utilize them as a source of carbon/nitrogen, energy, and electrons with dynamic metabolic diversity. This could serve as an eco-friendly alternative to mitigate their toxicity. For example, the bacterium, *Bacillus cereus* H38, has been shown to possess two pathways, *i.e.*, S-N bond cleavage and N^4^-amine cleavage for sulfonamide degradation, and *Pseudomonas psychrophila* HA-4 for sulfamethoxazole (hydroxy and amino-benzenesulfonamide) degradation. The involvement of inducible enzymes, such as flavin-dependent monooxygenases, FMN reductase, and 1,4-benzoquinone reductase, has been reported in a wide variety of taxa. The role of the consortium (XG) consisting of species of *Achromobacter*, *Bacillus*, *Lactococcus*, *Ochrobactrum*, and *Enterococcus* has been reported in the mineralization of other antibiotics. For analgesics, *Sphingomonas* sp. Ibu-2 has been reported to convert ibuprofen to ibuprofen-CoA via the action of a CoA-ligase. This is followed by the formation of isobutylcatechol, with subsequent *meta*-ring cleavage. On the other hand, *Variovorax* sp. Ibu-1 has been proven to metabolize ibuprofen via the formation of trihydroxyibuprofen, which then undergoes *meta*-ring cleavage to form aliphatic intermediates mediated by the *ipf*ABDEF gene cluster. Interestingly, *Sphingopyxis-*mediated clearance (through the formation of phenylacetic acid derivatives) of harmful algal blooms (due to eutrophication), which release cyanotoxins (microcystins and nodularin: complex species of cyclic penta- and hexapeptides), has been highlighted. In the case of plasticizers (phthalate ester isomers of mid and long chain) which have been reported as potent endocrine-disrupting chemicals (EDCs), metabolism by *Gordonia*, *Rhodococcus*, *Pseudomonas*, *Cupravidus*, *Burkholderia*, *Achromobacter*, *Agromyces*, *Microbacterium*, *Acinetobacter*, and *Bacillus* has been reported. Initial degradation occurs through either a) de-esterification leading to the formation of mono-alkyl esters such as the conversion of DEHP to mono-(2-ethylhexyl) phthalate (MEHP), and then further to phthalic acid, or b) stepwise beta-oxidation of the alkyl side chains such as the conversion through intermediates like diethyl phthalate (DEP), mono-methyl phthalate (MMP), or butyl methyl phthalate (BMP), which ultimately funnels into central carbon pathways. The metabolic routes and enzymes involved in the degradation of the most commonly used benzene-based pesticides (imidacloprid and chlorpyrifos) and the herbicide (glyphosate) have also been underlined, with special mention of metal-dependent enzymes such as hydrolases (organophosphorus hydrolase, phosphotriesterase, methyl parathion hydrolase and organophosphorus acid anhydrolase), thus reducing toxicity and activating compounds for better degradation. The importance of multi-omics approaches such as genomics in conjunction with transcriptomics and proteomics to identify the up/downregulation of genes/proteins under target conditions has been emphasized. Considering the limitations *(*slow degradation rates, incomplete transformation, reduced survivability, *etc*.) of using bacterial strains/cultures/consortia at the site, the use of directed genetic engineering, or “metabolic engineering” has been proposed to enhance the metabolic diversity, degradation rates, and physiological vigor, and to overcome carbon catabolite repression*,* thus offering potential research opportunities.

Persistent oil hydrocarbons (OHs, which include alkanes and aromatics and their substituted *n-mers*) discharged from spillage, refinery extraction, pipelines, and service stations mostly lead to soil, food, and groundwater contamination. In this Research Topic, Melzi et al. highlighted the use of phytoremediation (microbe-assisted) as an ideal solution to eliminate or reduce the concentration of OHs. The authors presented an example of a phytoremediation-based bioremediation intervention for OHs (both C ≤ 12 and C > 12) that were spilled from an oil spillage site to an aquifer and the wetland soil containing *Scirpus sylvaticus* (L.). The prevalence of diatoms (*Ulnaria* spp.) was identified as a bioindicator species. A soil microcosm examination indicated a reduction in OH phytotoxicity and phytodegradation (up to 82%) when treated with *Zea mays* and *Helianthus annuus*. It has also been demonstrated that the involvement of functional genes encoding toluene-benzene monooxygenase (*tbm*D) and alkane hydroxylase (*alk*B) enabled the community to achieve higher biodegradation rates, thus indicating a natural bio-attenuation process and a possible application for site recovery.

In light of the high energy demand and dependency on a carbon-neutral economy, better bio-prospecting of coal mines and their waste/waste coal requires major attention. Coalbed methane (CBM) has emerged as an important energy source in developing nations such as India and it is expected to play a significant role in the energy portfolio of the future. Basera et al. reported enhanced methane production from lignite (the lowest-rank coal) through optimization of bioconversion to methane at a temperature of 55°C and an NaCl concentration of 0.15%. A scale-up study demonstrated a higher methane yield (2,800 mM of methane per 25 g of lignite) under anaerobic conditions. The bioconversion route and intermediates were confirmed through Fourier transform infrared (FTIR) spectroscopy and gas chromatography-mass spectrometry (GC-MS). The authors highlighted that a bacterial consortium converted lignite into volatile fatty acids, which were subsequently converted into methane by *Methanosarcinales* and *Methanomicrobiales*. Thus, the study showed the potential for developing indigenous consortia that could potentially enhance methane production from the low-rank coal in Indian coal beds under thermophilic conditions.

The discharge of radioactive waste from nuclear power plants, nuclear weapons testing, and medical procedures releases dangerous radioactive iodine isotopes into the environment. Duborska et al. highlighted the importance of eliminating iodine radionuclides (36 known radioactive isotopes of iodine, including ^129^I and ^131^I, which mainly occur as iodate) from polluted environments. Although physicochemical methods (metal oxide chelation, bentonite clay adsorption, and biomass composite) have been attempted, microbial iodine speciation/transformation and removal have gained attention. The authors reviewed iodate reduction by non-specific nitrate and chlorate reductase and iodate reductase by metal-interacting bacterial species *such as Shewanella* spp. and *Agrobacterium* spp., and reported on the recruitment of key metabolites such as glutathione. *Shewanella oneidensis* has been shown to reduce iodate extracellularly by employing the outer membrane MtrAB and membrane-bound dimethyl sulfoxide (DMSO) reductase with a molybdenum enzyme center that acts as a catalytic site. Strains of *Roseobacter* and *Rhodothalassium* have been reported to oxidize iodine species by employing a multi-copper oxidase system. Fungi such as *Aspergillus*, which employ an oxidase to transform iodine have also been investigated. Volatilization to organic iodines (methylated forms) has also been reported in *Proteobacteria*, *Cytophaga*-*Flexibacter*-*Bacteroides* and some cyanobacteria and algal species, but the molecular details have yet to be confirmed. With higher uptake rates in microbes such as *Ralstonia*, *Cupriavidus*, *Bacillus*, *Streptomyces*, *etc.,* these organisms bioaccumulate iodine into various biomass fractions, which could be explored for the industrial extraction of iodine from saline and brine systems. Additionally, Duborska et al. highlighted the use of biomass-derived composites such as cellulose nanocomposites and organic frameworks with metals (Zn), for sustainable iodine recovery and remediation efforts. This observation is in line with the use of bio-transforming/redox-active microbes combined with plants (phytoremediation) for the successful bioremediation of iodine, cesium and uranium from contaminated sites (Thakur and Kumar, 2024).

Finally, we believe that the Research Topic on “Xenobiotic and Emerging Contaminants in Ecosystems” will provide key insights into recent advances in the use of microbial inoculants, bioprocesses, and climate-smart biotechnologies to remediate contaminated sites while preserving ecosystem health. It will also help researchers and policymakers to rationalize effective and innovative cleanup processes, thus reducing the potential risk of exposure to these contaminants.
